# Supplementary material for: Treatment Outcomes of Novel Targeted Agents in Relapse/Refractory Chronic Lymphocytic Leukemia: A Systematic Review and Network Meta-Analysis
Source: J Clin Med. 2019 May 23;8(5):737. doi: 10.3390/jcm8050737 (PMC6572611; doi:10.3390/jcm8050737)
Supplement: Supplementary file 1 [file jcm-08-00737-s001.pdf]

Supplementary appendix

Treatment Outcomes of Novel Targeted Agents in Relapse or Refractory Chronic Lymphocytic Leukemia: A Systematic Literature Review and Network Meta-analysis

Po-Huang Chen M.D., Ching-Liang Ho M.D. PhD., Chin Lin PhD., Yi-Ying Wu M.D. PhD., Tzu-Chuan Huang M.D., Yu-Kang Tu PhD.[#], Cho-Hao Lee M.D.*

Contents

Supplementary Appendix 1. Protocol for the present systematic review

Supplementary Appendix 2. PRISMA checklist

Supplementary Appendix 3. Search details

Supplementary Appendix 4. list of included full-text screening studies

Supplementary Appendix 5. Risk of bias

Supplementary Appendix 6. Efficacy Outcomes for Progression Free Survival (PFS) and Overall

Survival (OS) in Network Meta-analysis: Head-to-head comparisons of outcomes

Appendix 1 Review Protocol

This mixed methods systematic review is registered with the International Prospective Register of Systematic Reviews (PROSPERO): CRD42018088179

PROSPERO:https://www.crd.york.ac.uk/prospero/

Appendix 2 PRIMSA checklist

Section/topic	Item No	Checklist item	Reported on page No
**Title**			
Title	1	Identify the report as a systematic review, meta-analysis, or both	1
**Abstract**			
Structured summary	2	Provide a structured summary including, as applicable, background, objectives, data sources, study eligibility criteria, participants, interventions, study appraisal and synthesis methods, results, limitations, conclusions and implications of key findings, systematic review registration number	4-5
**Introduction**			
Rationale	3	Describe the rationale for the review in the context of what is already known	6-8
Objectives	4	Provide an explicit statement of questions being addressed with reference to participants, interventions, comparisons, outcomes, and study design (PICOS)	6-8
**Methods**			
Protocol and registration	5	Indicate if a review protocol exists, if and where it can be accessed (such as web address), and, if available, provide registration information including registration number	9
Eligibility criteria	6	Specify study characteristics (such as PICOS, length of follow-up) and report characteristics (such as years considered, language, publication status) used as criteria for eligibility, giving rationale	9
Information sources	7	Describe all information sources (such as databases with dates of coverage, contact with study authors to identify additional studies) in the search and date last searched	9
Search	8	Present full electronic search strategy for at least one database, including any limits used, such that it could be repeated	Appendices
Study selection	9	State the process for selecting studies (that is, screening, eligibility, included in systematic review, and, if applicable, included in the meta-analysis)	9
Data collection process	10	Describe method of data extraction from reports (such as piloted forms, independently, in duplicate) and any processes for obtaining and confirming data from investigators	10
Data items	11	List and define all variables for which data were sought (such as PICOS, funding sources) and any assumptions and simplifications made	10

Section/topic	Item No	Checklist item	Reported on page No
Risk of bias in individual studies	12	Describe methods used for assessing risk of bias of individual studies (including specification of whether this was done at the study or outcome level), and how this information is to be used in any data synthesis	11
Summary measures	13	State the principal summary measures (such as risk ratio, difference in means).	10-11
Synthesis of results	14	Describe the methods of handling data and combining results of studies, if done, including measures of consistency (such as I^2 statistic) for each meta-analysis	10-11
Risk of bias across studies	15	Specify any assessment of risk of bias that may affect the cumulative evidence (such as publication bias, selective reporting within studies)	10-11
Additional analyses	16	Describe methods of additional analyses (such as sensitivity or subgroup analyses, meta-regression), if done, indicating which were pre-specified	10-11
**Results**			
Study selection	17	Give numbers of studies screened, assessed for eligibility, and included in the review, with reasons for exclusions at each stage, ideally with a flow diagram	12, Figure 1
Study characteristic s	18	For each study, present characteristics for which data were extracted (such as study size, PICOS, follow-up period) and provide the citations	12, Table 1
Risk of bias within studies	19	Present data on risk of bias of each study and, if available, any outcome-level assessment (see item 12).	12, Appendices
Results of individual studies	20	For all outcomes considered (benefits or harms), present for each study (a) simple summary data for each intervention group and (b) effect estimates and confidence intervals, ideally with a forest plot	12-14, Table 2
Synthesis of results	21	Present results of each meta-analysis done, including confidence intervals and measures of consistency	10-12, Figures 1-2; Table 2
Risk of bias across studies	22	Present results of any assessment of risk of bias across studies (see item 15)	10-12, Figures 1-2; Table 2

Section/topic	Item No	Checklist item	Reported on page No
Additional analysis	23	Give results of additional analyses, if done (such as sensitivity or subgroup analyses, meta-regression) (see item 16)	Appendices
**Discussion**			
Summary of evidence	24	Summarise the main findings including the strength of evidence for each main outcome; consider their relevance to key groups (such as health care providers, users, and policy makers)	13-15
Limitations	25	Discuss limitations at study and outcome level (such as risk of bias), and at review level (such as incomplete retrieval of identified research, reporting bias)	15
Conclusions	26	Provide a general interpretation of the results in the context of other evidence, and implications for future research	16
**Funding**			
Funding	27	Describe sources of funding for the systematic review and other support (such as supply of data) and role of funders for the systematic review	None

Appendix 3 Literature search strategy

**Relevant text of Population**	**Relevant text of intervention**
1.B cell Leukemia	1.Target therapy
2.Lymphocytic leukemia	2.Chemoimmunotherapy
3.Chronic lymphocytic leukemia	3.Small-molecule inhibitor
4.Small lymphocytic leukemia	4.Bruton's tyrosine kinase inhibitor
A= #1 or #2 or #3 or #4	5.Ibrutinib
1.Recurrent	6.Imbruvica
2.Refractory	7.Phosphoinositide 3 kinase δ inhibitor
3.Relapse	8.Idelalisib
B = #1 or #2 or #3	9.Zydelig
4.Progression free survival	10.Venetoclax
5.Overall Survival	11.Venclexta
6.Response	12.Duvelisib
C = #1 or #2 or #3	13.Copiktra
7.Trial	14.Bendamustine
8.Clinical trial/ trials	15.Innomustine
9.Randomized	16.Ofatumumab
10.Randomization	17.Campathve
11.Controlled trial/trials	18.Rituximab
12.Randomized controlled	19.Rituxan
trial/trials	E = #1 or #2 or #3 or #4 or #5 or #6 or
13.Controlled clinical trial	#7 or #8 or #9 or #10 or #11 or #12 or
14.RCT	#13 or #14 or #15 or #16 or #17 or #18
15.Perspective study	or #19

D = #1 or #2 or #3 or #4 or #5 or #6 or #7 or #8 or #9 or #20 or #21	*. All search keyword with [Mesh Terms] or [All Fields]

PUBMED: http://www.ncbi.nlm.nih.gov/pubmed
EMBASE: https://www.embase.com
COCHRANE CENTRAL: https://www.cochrane.com

Search	Query	Items found
Population#1	Search (((B cell Leukemia[Title/Abstract]) OR Lymphocytic leukemia[Title/Abstract]) OR Chronic lymphocytic leukemia[Title/Abstract]) OR Small lymphocytic leukemia[Title/Abstract]	21380
Population#2	Search ((Recurrent[Title/Abstract]) OR Refractory[Title/Abstract]) OR Relapse[Title/Abstract]	468169
Population	Search (((((B cell Leukemia[Title/Abstract]) OR Lymphocytic leukemia[Title/Abstract]) OR Chronic lymphocytic leukemia[Title/Abstract]) OR Small lymphocytic leukemia[Title/Abstract])) AND (((Recurrent[Title/Abstract]) OR Refractory[Title/Abstract]) OR Relapse[Title/Abstract])	2526
Intervention	Search (((((((((((((((((((Target therapy[Title/Abstract]) OR Chemoimmunotherapy[Title/Abstract]) OR Small-molecule inhibitor[Title/Abstract]) OR Bruton's tyrosine kinase inhibitor[Title/Abstract]) OR Ibrutinib[Title/Abstract]) OR Imbruvica[Title/Abstract]) OR Phosphoinositide 3 kinase δ inhibitor[Title/Abstract]) OR Idelalisib[Title/Abstract]) OR Zydelig[Title/Abstract]) OR Venetoclax[Title/Abstract]) OR Venclexta[Title/Abstract]) OR Duvelisib[Title/Abstract]) OR Copiktra[Title/Abstract]) OR Bendamustine[Title/Abstract]) OR Innomustine[Title/Abstract]) OR Ofatumumab[Title/Abstract]) OR Arzerra[Title/Abstract]) OR Alemtuzumab[Title/Abstract]) OR Campathve[Title/Abstract]) OR Rituximab[Title/Abstract]) OR Rituxan[Title/Abstract]	29441
type	Search ((((((((((Trial[Title/Abstract]) OR Clinical trial[Title/Abstract]) OR Randomized[Title/Abstract]) OR Randomization[Title/Abstract]) OR Randomized controlled trial[Title/Abstract]) OR Controlled trial[Title/Abstract]) OR Controlled clinical trial[Title/Abstract]) OR RCT[Title/Abstract]) OR Perspective study[Title/Abstract]) OR Clinical study[Title/Abstract]) OR Clinical article[Title/Abstract]	843944
#7	Search (((((((B cell Leukemia[Title/Abstract]) OR Lymphocytic leukemia[Title/Abstract]) OR Chronic lymphocytic leukemia[Title/Abstract]) OR Small lymphocytic leukemia[Title/Abstract])) AND (((Recurrent[Title/Abstract]) OR Refractory[Title/Abstract]) OR Relapse[Title/Abstract]))) AND (((((((((((Trial[Title/Abstract]) OR Clinical trial[Title/Abstract]) OR Randomized[Title/Abstract]) OR Randomization[Title/Abstract]) OR Randomized controlled trial[Title/Abstract]) OR Controlled trial[Title/Abstract]) OR Controlled clinical trial[Title/Abstract]) OR RCT[Title/Abstract]) OR Perspective study[Title/Abstract]) OR Clinical study[Title/Abstract]) OR Clinical article[Title/Abstract])	357

#5	Search ((((((((((((((((((((((Target therapy[Title/Abstract]) OR Chemoimmunotherapy[Title/Abstract]) OR Small-molecule inhibitor[Title/Abstract]) OR Bruton's tyrosine kinase inhibitor[Title/Abstract]) OR Ibrutinib[Title/Abstract]) OR Imbruvica[Title/Abstract]) OR Phosphoinositide 3 kinase δ inhibitor[Title/Abstract]) OR Idelalisib[Title/Abstract]) OR Zydelig[Title/Abstract]) OR Venetoclax[Title/Abstract]) OR Venclexta[Title/Abstract]) OR Duvelisib[Title/Abstract]) OR Copiktra[Title/Abstract]) OR Bendamustine[Title/Abstract]) OR Innomustine[Title/Abstract]) OR Ofatumumab[Title/Abstract]) OR Arzerra[Title/Abstract]) OR Alemtuzumab[Title/Abstract]) OR Campathve[Title/Abstract]) OR Rituximab[Title/Abstract]) OR Rituxan[Title/Abstract])) AND (((((((((((Trial[Title/Abstract]) OR Clinical trial[Title/Abstract]) OR Randomized[Title/Abstract]) OR Randomization[Title/Abstract]) OR Randomized controlled trial[Title/Abstract]) OR Controlled trial[Title/Abstract]) OR Controlled clinical trial[Title/Abstract]) OR RCT[Title/Abstract]) OR Perspective study[Title/Abstract]) OR Clinical study[Title/Abstract]) OR Clinical article[Title/Abstract])) AND (((Recurrent[Title/Abstract]) OR Refractory[Title/Abstract]) OR Relapse[Title/Abstract])) AND ((((B cell Leukemia[Title/Abstract]) OR Lymphocytic leukemia[Title/Abstract]) OR Chronic lymphocytic leukemia[Title/Abstract]) OR Small lymphocytic leukemia[Title/Abstract])	289

EMBASE

(**'b cell leukemia'**/exp OR **'lymphatic leukemia'**/exp OR **'chronic lymphatic leukemia'**/exp OR **'small lymphocytic leukemia'**/exp) AND (**'target therapy'** OR **'chemoimmunotherapy'**/exp OR **'small molecule inhibitor'**/exp OR **'bruton tyrosine kinase inhibitor'**/exp OR **'ibrutinib'**/exp OR **'phosphatidylinositol 3 kinase inhibitor'**/exp OR **'idelalisib'**/exp OR **'venetoclax'**/exp OR **'duvelisib'**/exp OR **'bendamustine'**/exp OR **'ofatumumab'**/exp OR **'alemtuzumab'**/exp OR **'rituximab'**/exp) AND (**'recurrent disease'**/exp OR **refractory** OR **'relapse'**/exp) AND ([controlled clinical trial]/lim OR [randomized controlled trial]/lim)

Appendix 4 Reference list of full-text screening studies.

Bergmann, M. A., Goebeler, M. E., Herold, M., Emmerich, B., Wilhelm, M., Ruelfs, C., . . . Hallek, M. J. (2005). Efficacy of bendamustine in patients with relapsed or refractory chronic lymphocytic leukemia: Results of a phase I/II study of the German CLL Study Group. *Haematologica, 90*(10), 1357-1364. Retrieved from http://www.embase.com/search/results?subaction=viewrecord&from=export&id=L41503651

http://www.haematologica.org/content/haematol/90/10/1357.full.pdf

Coiffier, B., Lepretre, S., Pedersen, L. M., Gadeberg, O., Fredriksen, H., Van Oers, M. H. J., . . . Robak, T. (2008). Safety and efficacy of ofatumumab, a fully human monoclonal anti-CD20 antibody, in patients with relapsed or refractory B-cell chronic lymphocytic leukemia: A phase 1-2 study. *Blood, 111*(3), 1094-1100. doi:10.1182/blood-2007-09-111781

Blum, K. A., Advani, A., Fernandez, L., Van Der Jagt, R., Brandwein, J., Kambhampati, S., . . . Byrd, J. C. (2009). Phase II study of the histone deacetylase inhibitor MGCD0103 in patients with previously treated chronic lymphocytic leukaemia. *British Journal of Haematology, 147*(4), 507-514. doi:10.1111/j.1365-2141.2009.07881.x

Lin, T. S., Naumovski, L., Lecane, P. S., Lucas, M. S., Moran, M. E., Cheney, C., . . . Byrd, J. C. (2009). Effects of motexafin gadolinium in a phase II trial in refractory chronic lymphocytic leukemia. *Leuk Lymphoma, 50*(12), 1977-1982. doi:10.3109/10428190903288464

O'Brien, S., Moore, J. O., Boyd, T. E., Larratt, L. M., Skotnicki, A. B., Koziner, B., . . . Rai, K. R. (2009). 5-year survival in patients with relapsed or refractory chronic lymphocytic leukemia in a randomized, phase III trial of fludarabine plus cyclophosphamide with or without oblimersen. *J Clin Oncol, 27*(31), 5208-5212. doi:10.1200/jco.2009.22.5748

Dretzke, J., Barton, P., Kaambwa, B., Connock, M., Uthman, O., Bayliss, S., & Meads, C. (2010). Rituximab for the treatment of relapsed/refractory chronic lymphocytic leukaemia. *Health Technol Assess, 14*(Suppl. 2), 19-26. doi:10.3310/hta14suppl2/03

Wierda, W. G., Kipps, T. J., Mayer, J., Stilgenbauer, S., Williams, C. D., Hellmann, A., . . . Osterborg, A. (2010). Ofatumumab as single-agent CD20 immunotherapy in fludarabine-refractory chronic lymphocytic leukemia. *J Clin Oncol, 28*(10), 1749-1755. doi:10.1200/jco.2009.25.3187

Amrein, P. C., Attar, E. C., Takvorian, T., Hochberg, E. P., Ballen, K. K., Leahy, K. M., . . . Brown, J. R. (2011). Phase II study of dasatinib in relapsed or refractory chronic lymphocytic leukemia. *Clin Cancer Res, 17*(9), 2977-2986. doi:10.1158/1078-0432.ccr-10-2879

Badoux, X. C., Keating, M. J., Wang, X., O'Brien, S. M., Ferrajoli, A., Faderl, S., . . . Wierda, W. G. (2011). Fludarabine, cyclophosphamide, and rituximab chemoimmunotherapy is highly effective treatment for relapsed patients with CLL. *Blood, 117*(11), 3016-3024. doi:10.1182/blood-2010-08-304683

Bezares, R. F., Stemelin, G., Diaz, A., Argentieri, D., Zubiaur, E. L., Garay, G., . . . Milone, G. (2011). Multicenter study of subcutaneous alemtuzumab administered at reduced dose in patients with fludarabine-relapsed/refractory chronic lymphocytic leukemia: final analysis. *Leuk Lymphoma, 52*(10), 1936-1941. doi:10.3109/10428194.2011.584991

Elter, T., Gercheva-Kyuchukova, L., Pylylpenko, H., Robak, T., Jaksic, B., Rekhtman, G., . . . Engert, A. (2011). Fludarabine plus alemtuzumab versus fludarabine alone in patients with previously treated chronic lymphocytic leukaemia: A randomised phase 3 trial. *The Lancet Oncology, 12*(13), 1204-1213. doi:10.1016/S1470-2045(11)70242-X

Fischer, K., Cramer, P., Busch, R., Stilgenbauer, S., Bahlo, J., Schweighofer, C. D., . . . Wendtner, C. M. (2011). Bendamustine combined with rituximab in patients with relapsed and/or refractory chronic lymphocytic leukemia: a multicenter phase II trial of the German Chronic Lymphocytic Leukemia Study Group. *J Clin Oncol, 29*(26), 3559-3566. doi:10.1200/jco.2010.33.8061

Hillmen, P., Cohen, D. R., Cocks, K., Pettitt, A., Sayala, H. A., Rawstron, A. C., . . . Pocock, C. (2011). A randomized phase II trial of fludarabine, cyclophosphamide and mitoxantrone (FCM) with or without rituximab in previously treated chronic lymphocytic leukaemia. *British Journal of Haematology, 152*(5), 570-578. doi:10.1111/j.1365-2141.2010.08317.x

Wierda, W. G., Padmanabhan, S., Chan, G. W., Gupta, I. V., Lisby, S., & Österborg, A. (2011). Ofatumumab is active in patients with fludarabine-refractory CLL irrespective of prior rituximab: Results from the phase 2 international study. *Blood, 118*(19), 5126-5129. doi:10.1182/blood-2011-04-348656

Montillo, M. (2012). Is bendamustine an ideal partner for rituximab in the management of relapsed chronic lymphocytic leukemia? Results of a multicenter Phase II trial. *Expert Rev Hematol, 5*(1), 43-46. doi:10.1586/ehm.11.75

Roberts, A. W., Seymour, J. F., Brown, J. R., Wierda, W. G., Kipps, T. J., Khaw, S. L., . . . Humerickhouse, R. (2012). Substantial susceptibility of chronic lymphocytic leukemia to BCL2 inhibition: results of a phase I study of navitoclax in patients with relapsed or refractory disease. *J Clin Oncol, 30*(5), 488-496. doi:10.1200/jco.2011.34.7898

Smolej, L., Doubek, M., Panovska, A., Simkovic, M., Brychtova, Y., Belada, D., . . . Mayer, J. (2012). Rituximab in combination with high-dose dexamethasone for the treatment of relapsed/refractory chronic lymphocytic leukemia. *Leuk Res, 36*(10), 1278-1282. doi:10.1016/j.leukres.2012.07.005

Burotto, M., Stetler-Stevenson, M., Arons, E., Zhou, H., Wilson, W., & Kreitman, R. J. (2013). Bendamustine and rituximab in relapsed and refractory hairy cell leukemia. *Clinical Cancer Research, 19*(22), 6313-6321. doi:10.1158/1078-0432.CCR-13-1848

Byrd, J. C., Barrientos, J. C., Devereux, S., Brown, J. R., Kay, N. E., Reddy, N. M., . . . Hillmen, P. (2013). A randomized, multicenter, open-label, phase III study of the Bruton tyrosine kinase (BTK) inhibitor ibrutinib (PCI-32765) versus ofatumumab in patients (pts) with relapsed or refractory (RR) chronic lymphocytic leukemia (CLL)/small lymphocytic lymphoma (SLL): RESONATE. *Journal of Clinical Oncology, 31*(15). Retrieved from http://www.embase.com/search/results?subaction=viewrecord&from=export&id=L71102341

Eradat, H. A., Coutre, S. E., Barrientos, J. C., Rai, K. R., Farber, C. M., Hillmen, P., . . . Hallek, M. J. (2013). A phase III, randomized, double-blind, placebo-controlled study evaluating the efficacy and safety of idelalisib (GS-1101) in combination with bendamustine and rituximab for previously treated chronic

lymphocytic leukemia (CLL). *Journal of Clinical Oncology, 31*(15). Retrieved from http://www.embase.com/search/results?subaction=viewrecord&from=export&id=L71102317

Ogawa, Y., Ogura, M., Suzuki, T., Ando, K., Uchida, T., Shirasugi, Y., . . . Hotta, T. (2013). A phase I/II study of ofatumumab (GSK1841157) in Japanese and Korean patients with relapsed or refractory B-cell chronic lymphocytic leukemia. *Int J Hematol, 98*(2), 164-170. doi:10.1007/s12185-013-1393-x

Tsimberidou, A. M., Wierda, W. G., Wen, S., Plunkett, W., O'Brien, S., Kipps, T. J., . . . Keating, M. J. (2013). Phase I-II clinical trial of oxaliplatin, fludarabine, cytarabine, and rituximab therapy in aggressive relapsed/refractory chronic lymphocytic leukemia or Richter syndrome. *Clin Lymphoma Myeloma Leuk, 13*(5), 568-574. doi:10.1016/j.clml.2013.03.012

Byrd, J. C., Brown, J. R., O'Brien, S., Barrientos, J. C., Kay, N. E., Reddy, N. M., . . . Hillmen, P. (2014). Ibrutinib versus Ofatumumab in Previously Treated Chronic Lymphoid Leukemia. *New England Journal of Medicine, 371*(3), 213-223. doi:10.1056/NEJMoa1400376

Cortelezzi, A., Sciume, M., Liberati, A. M., Vincenti, D., Cuneo, A., Reda, G., . . . Foa, R. (2014). Bendamustine in combination with ofatumumab in relapsed or refractory chronic lymphocytic leukemia: a GIMEMA Multicenter Phase II Trial. *Leukemia, 28*(3), 642-648. doi:10.1038/leu.2013.334

Fabre, C., Gobbi, M., Ezzili, C., Zoubir, M., Sablin, M. P., Small, K., . . . Le Tourneau, C. (2014). Clinical study of the novel cyclin-dependent kinase inhibitor dinaciclib in combination with rituximab in relapsed/refractory chronic lymphocytic leukemia patients. *Cancer Chemother Pharmacol, 74*(5), 1057-1064. doi:10.1007/s00280-014-2583-9

Kater, A. P., Spiering, M., Liu, R. D., Doreen te Raa, G., Slinger, E., Tonino, S. H., . . . van Oers, M. H. J. (2014). Dasatinib in combination with fludarabine in patients with refractory chronic lymphocytic leukemia: A multicenter phase 2 study. *Leuk Res, 38*(1), 34-41. doi:10.1016/j.leukres.2013.10.004

Doubek, M., Brychtova, Y., Panovska, A., Sebejova, L., Stehlikova, O., Chovancova, J., . . . Mayer, J. (2015). Ofatumumab added to dexamethasone in patients with relapsed or refractory chronic lymphocytic leukemia: Results from a phase II study. *Am J Hematol, 90*(5), 417-421. doi:10.1002/ajh.23964

Flowers, C. R., Brown, J. R., Rosenthal, H., Stock, W., Katzen, H. I., Cohen, J. B., . . . Jaye, D. L. (2015). A Phase 2 Trial of Fludarabine Combined With Subcutaneous Alemtuzumab for the Treatment of Relapsed/Refractory B-Cell Chronic Lymphocytic Leukemia. *Clin Lymphoma Myeloma Leuk, 15*(11), 694-698. doi:10.1016/j.clml.2015.07.640

Hillmen, P., Fraser, G., Jones, J., Rule, S., Brien, S., Dilhuydy, M. S., . . . Chanan-Khan, A. (2015). Comparing Single-Agent Ibrutinib, Bendamustine Plus Rituximab (BR) and Ibrutinib Plus BR in Patients with Previously Treated Chronic Lymphocytic Leukemia/Small Lymphocytic Lymphoma (CLL/SLL): An Indirect Comparison of the RESONATE and HELIOS Trials. *Blood, 126*(23), 2944. Retrieved from http://www.bloodjournal.org/content/126/23/2944.abstract

Jones, J. A., Wach, M., Robak, T., Brown, J. R., Menter, A. R., Vandenberghe, E., . . . Owen, C. (2015). Results of a phase III randomized, controlled study evaluating the efficacy and safety of idelalisib (IDELA) in combination with ofatumumab (OFA) for previously treated chronic lymphocytic leukemia

(CLL). *Journal of Clinical Oncology, 33*(15). Retrieved from http://www.embase.com/search/results?subaction=viewrecord&from=export&id=L72014362

Lanasa, M. C., Andritsos, L., Brown, J. R., Gabrilove, J., Caligaris-Cappio, F., Ghia, P., . . . Grever, M. R. (2015). Final results of EFC6663: A multicenter, international, phase 2 study of alvocidib for patients with fludarabine-refractory chronic lymphocytic leukemia. *Leuk Res, 39*(5), 495-500. doi:10.1016/j.leukres.2015.02.001

Larsen, J. T., Shanafelt, T. D., Leis, J. F., LaPlant, B. R., Call, T. G., Zent, C. S., . . . Ding, W. (2015). The Akt inhibitor MK-2206 in combination with rituximab and bendamustine demonstrates efficacy in relapsed/refractory chronic lymphocytic leukemia: Updated results from the NCCTG N1087 Alliance study. *Molecular Cancer Therapeutics, 14*(7). doi:10.1158/1538-8514.PI3K14-B02

Ujjani, C., Ramzi, P., Gehan, E., Wang, H., Wang, Y., & Cheson, B. D. (2015). Ofatumumab and bendamustine in previously treated chronic lymphocytic leukemia and small lymphocytic lymphoma. *Leukemia and Lymphoma, 56*(4), 915-920. doi:10.3109/10428194.2014.933217

Yang, Q., Modi, P., Newcomb, T., Queva, C., & Gandhi, V. (2015). Idelalisib: First-in-Class PI3K Delta Inhibitor for the Treatment of Chronic Lymphocytic Leukemia, Small Lymphocytic Leukemia, and Follicular Lymphoma. *Clin Cancer Res, 21*(7), 1537-1542. doi:10.1158/1078-0432.Ccr-14-2034

Anderson, M. A., Roberts, A. W., Seymour, J. F., Gerecitano, J. F., Kahl, B. S., Kipps, T. J., . . . Davids, M. S. (2016). Safety, efficacy and immune effects of venetoclax 400 mg daily in patients (PTS) with relapsed chronic lymphocytic leukemia (CLL). *Haematologica, 101*, 228. Retrieved from http://www.embase.com/search/results?subaction=viewrecord&from=export&id=L615550547

Brien, S. M., Furman, R. R., Coutre, S. E., Flinn, I. W., Burger, J., Blum, K., . . . Byrd, J. C. (2016). Five-Year Experience with Single-Agent Ibrutinib in Patients with Previously Untreated and Relapsed/Refractory Chronic Lymphocytic Leukemia/Small Lymphocytic Leukemia. *Blood, 128*(22), 233. Retrieved from http://www.bloodjournal.org/content/128/22/233.abstract

Brown, J. R., Harb, W. A., Hill, B. T., Gabrilove, J., Sharman, J. P., Schreeder, M. T., . . . Kipps, T. J. (2016). Phase I study of single-agent CC-292, a highly selective bruton’s tyrosine kinase inhibitor, in relapsed/refractory chronic lymphocytic leukemia. *Haematologica, 101*(7), e295-e298. doi:10.3324/haematol.2015.140806

Chanan-Khan, A., Cramer, P., Demirkan, F., Fraser, G., Silva, R. S., Grosicki, S., . . . Hallek, M. (2016). Ibrutinib combined with bendamustine and rituximab compared with placebo, bendamustine, and rituximab for previously treated chronic lymphocytic leukaemia or small lymphocytic lymphoma (HELIOS): a randomised, double-blind, phase 3 study. *Lancet Oncol, 17*(2), 200-211. doi:10.1016/s1470-2045(15)00465-9

Chavez, J. C., Piris-Villaespesa, M., Dalia, S., Powers, J., Turba, E., Nodzon, L., . . . Pinilla-Ibarz, J. (2016). Results of a phase II study of lenalidomide and rituximab for refractory/relapsed chronic lymphocytic leukemia. *Leuk Res, 47*, 78-83. doi:10.1016/j.leukres.2016.05.012

Coutre, S., Wierda, W., Choi, M., Davids, M. S., Cheson, B. D., Furman, R. R., . . . Jones, J. (2016). Venetoclax is active in CLL patients who have relapsed after or are refractory to ibrutinib or idelalisib.

Haematologica, 101, 231-232. Retrieved from http://www.embase.com/search/results?subaction=viewrecord&from=export&id=L615548718

Davids, M. S., Roberts, A. W., Seymour, J. F., Gerecitano, J. F., Kahl, B. S., Kipps, T. J., . . . Wierda, W. G. (2016). Safety, efficacy and immune effects of venetoclax 400 mg daily in patients with relapsed chronic lymphocytic leukemia (CLL). *Journal of Clinical Oncology, 34*. Retrieved from http://www.embase.com/search/results?subaction=viewrecord&from=export&id=L611752127

Doubek, M., Obrtlikova, P., Spacek, M., Urbanova, R., Diels, J., Thilakaratne, P., . . . Smolej, L. (2016). Single-agent ibrutinib vs standard of care for patients with relapsed/refractory (R/R) chronic lymphocytic leukemia (CLL): An adjusted comparison of resonate™ with the cllear database. *Haematologica, 101*, 230-231. Retrieved from http://www.embase.com/search/results?subaction=viewrecord&from=export&id=L615548623

Dreyling, M., Morschhauser, F., Bouabdallah, K., Cunningham, D., Bron, D., Linton, K., . . . Zinzani, P. L. (2016). Phase 2a study of the phosphatidylinositol-3-kinase (PI3K) inhibitor copanlisib in patients with relapsed/refractory, indolent or aggressive lymphoma. *Haematologica, 101*, 104. Retrieved from http://www.embase.com/search/results?subaction=viewrecord&from=export&id=L615549490

https://www.ncbi.nlm.nih.gov/pmc/articles/PMC4938333/pdf/1010104.pdf

Fischer, K., Al-Sawaf, O., Fink, A. M., Dixon, M., Bahlo, J., Warburton, S., . . . Hallek, M. (2016). Safety and efficacy of venetoclax and obinutuzumab in patients with previously untreated chronic lymphocytic leukemia (CLL) and coexisting medical conditions: Final results of the run-in phase of the randomized CLL14 trial (BO25323). *Blood, 128*(22). Retrieved from http://www.embase.com/search/results?subaction=viewrecord&from=export&id=L614310596

Jones, J., Choi, M. Y., Mato, A. R., Furman, R. R., Davids, M. S., Heffner, L. T., . . . Coutre, S. E. (2016). Venetoclax (VEN) monotherapy for patients with chronic lymphocytic leukemia (CLL) who relapsed after or were refractory to ibrutinib or idelalisib. *Blood, 128*(22). Retrieved from http://www.embase.com/search/results?subaction=viewrecord&from=export&id=L614225251

Jones, J. A., Robak, T., Wach, M., Brown, J. R., Menter, A. R., Vandenberghe, E., . . . Owen, C. (2016). Updated results of a phase III randomized, controlled study of idelalisib in combination with ofatumumab for previously treated chronic lymphocytic leukemia (CLL). *Journal of Clinical Oncology, 34*. Retrieved from http://www.embase.com/search/results?subaction=viewrecord&from=export&id=L611754113

Jones, J. A., Woyach, J., Awan, F. T., Maddocks, K. J., Whitlow, T., Ruppert, A. S., & Byrd, J. C. (2016). Phase 1b results of a phase 1b/2 study of obinutuzmab, ibrutinib, and venetoclax in relapsed/refractory chronic lymphocytic leukemia (CLL). *Blood, 128*(22). Retrieved from http://www.embase.com/search/results?subaction=viewrecord&from=export&id=L614225307

Leblond, V., Dilhuydy, M. S., Foà, R., Knauf, W., Montillo, M., Robinson, S., . . . Bosch, F. (2016). Preliminary safety data from the phase 3B green study of obinutuzumab (G) alone or combined with chemotherapy for previously untreated or relapsed/refractory chronic lymphocytic leukemia (CLL).

Haematologica, 101, 149. Retrieved from http://www.embase.com/search/results?subaction=viewrecord&from=export&id=L615549832

Portell, C. A., Chen, R. W., Wages, N., Cohen, J. B., Weber, M. J., Petroni, G., . . . Williams, M. E. (2016). Initial report of a multi-institution phase I/Ib study of ibrutinib with venetoclax in relapsed or refractory mantle cell lymphoma. *Blood, 128*(22). Retrieved from http://www.embase.com/search/results?subaction=viewrecord&from=export&id=L614247127

Robak, T., Jones, J., Wach, M., Brown, J., Menter, A., Vandenberghe, E., . . . Owen, C. (2016). Updated results of a phase 3 randomized, controlled study of idelalisib in combination with ofatumumab for previously treated chronic lymphocytic leukemia (CLL). *Haematologica, 101*, 53. Retrieved from http://www.embase.com/search/results?subaction=viewrecord&from=export&id=L615559305

Roberts, A. W., Seymour, J. F., Eichhorst, B., Stilgenbauer, S., Choi, M. Y., Davids, M. S., . . . Hallek, M. (2016). Pooled multi-trial analysis of venetoclax efficacy in patients with relapsed or refractory chronic lymphocytic leukemia. *Blood, 128*(22). Retrieved from http://www.embase.com/search/results?subaction=viewrecord&from=export&id=L614310315

Salles, G. A., Baseggio, L., Bachy, E., Sarkozy, C., Ghesquieres, H., Diels, J., . . . Callet-Bauchu, E. (2016). Single-agent ibrutinib vs standard of care for patients with relapsed/refractory (R/R) and treatment-naive (TN) chronic lymphocytic leukemia (CLL): An adjusted comparison of RESONATETM and RESONATE-2TM with the French lyon-sud database. *Blood, 128*(22). Retrieved from http://www.embase.com/search/results?subaction=viewrecord&from=export&id=L614310288

Sauter, C. S., Verwys, S. L., McCall, S. J., Miller, S. T., Courtien, A. I., Schoder, H., . . . Moskowitz, C. H. (2016). Phase i study combining ibrutinib with rituximab, ifosfamide, carboplatin, and etoposide (R-ICE) in patients with relapsed or primary refractory diffuse large B-cell lymphoma (DLBCL): NCI-cancer therapeutics evaluation program (CTEP) #9588. *Blood, 128*(22). Retrieved from http://www.embase.com/search/results?subaction=viewrecord&from=export&id=L614310118

Stilgenbauer, S., Eichhorst, B., Schetelig, J., Coutre, S., Seymour, J. F., Munir, T., . . . Wierda, W. G. (2016). Venetoclax in relapsed or refractory chronic lymphocytic leukaemia with 17p deletion: a multicentre, open-label, phase 2 study. *Lancet Oncol, 17*(6), 768-778. doi:10.1016/s1470-2045(16)30019-5

Stilgenbauer, S., Morschhauser, F., Wendtner, C. M., Cartron, G., Hallek, M., Eichhorst, B. F., . . . Salles, G. (2016). Phase ib study (GO28440) of venetoclax with bendamustine/rituximab or bendamustine/obinutuzumab in patients with relapsed/refractory or previously untreated chronic lymphocytic leukemia. *Blood, 128*(22). Retrieved from http://www.embase.com/search/results?subaction=viewrecord&from=export&id=L614309963

Wendtner, C. M., Byrd, J. C., Eichhorst, B., Foa, R., Hallek, M., Hillmen, P., . . . Woyach, J. A. (2016). A phase II study of MOR208 plus idelalisib in patients with relapsed or refractory chronic lymphocytic leukemia (CLL) or small lymphocytic lymphoma (SLL) previously treated with a Bruton's tyrosine kinase inhibitor (MIRACLE). *Journal of Clinical Oncology, 34*. Retrieved from http://www.embase.com/search/results?subaction=viewrecord&from=export&id=L611755419

Zelenetz, A., Robak, T., Coiffier, B., Delgado, J., Marlton, P., Adewoye, A., . . . Hillmen, P. (2016). Idelalisib plus bendamustine and rituximab (BR) is superior to BR alone in patients with relapsed/refractory chronic lymphocytic leukemia: Results of a phase 3 randomized double-blind placebo-controlled study. *British Journal of Haematology, 173*, 10. doi:10.1111/bjh.14019

Zent, C. S., Bowen, D. A., Conte, M. J., LaPlant, B. R., & Call, T. G. (2016). Treatment of relapsed/refractory chronic lymphocytic leukemia/small lymphocytic lymphoma with everolimus (RAD001) and alemtuzumab: a Phase I/II study. *Leuk Lymphoma, 57*(7), 1585-1591. doi:10.3109/10428194.2015.1113280

Anderson, M., Lew, T. E., Seymour, J. F., Roberts, A. W., & Tam, C. (2017). Outcomes with bruton tyrosine kinase inhibition in relapsed/refractory chronic lymphocytic leukemia/small lymphocytic lymphoma after progression on venetoclax. *Hematological Oncology, 35*, 236-237. doi:10.1002/hon.2438

Assouline, S., Amrein, L., Banerji, V., Caplan, S., Owen, C. J., Hasegawa, W., . . . Hay, A. E. (2017). A phase ii clinical trial of the pan pi3k inhibitor, buparlisib, for the treatment of relapsed and refractory chronic lymphocytic leukemia: Canadian cancer trials group IND.216. *Blood, 130*. Retrieved from http://www.embase.com/search/results?subaction=viewrecord&from=export&id=L620334191

Davids, M. S., Kim, H. T., Nicotra, A., Savell, A., Francoeur, K., Hellman, J. M., . . . Brown, J. R. (2017). Updated results of a multicenter phase I/Ib study of TGR-1202 in combination with ibrutinib in patients with relapsed or refractory MCL or CLL. *Hematological Oncology, 35*, 54-55. doi:10.1002/hon.2437

Davids, M. S., Savell, A., Kim, H. T., Abramson, J. S., Francoeur, K., Santiago-Medero, C., . . . Brown, J. R. (2017). Initial results of a phase IB study of ibrutinib in combination with obinutuzumab in patients with relapsed or refractory chronic lymphocytic leukemia. *Blood, 130*. Retrieved from http://www.embase.com/search/results?subaction=viewrecord&from=export&id=L620334162

Flinn, I. W., Hillmen, P., Montillo, M., Nagy, Z., Illés, Á., Etienne, G., . . . Stilgenbauer, S. (2017). Results from the phase 3 duo trial: A randomized comparison of duvelisib vs ofatumumab in patients with relapsed/refractory chronic lymphocytic leukemia or small lymphocytic lymphoma. *Blood, 130*. Retrieved from http://www.embase.com/search/results?subaction=viewrecord&from=export&id=L620311161

Garcia-Marco, J., Tello, P. B., Garcia, E. G., Herranz, E. R., Payer, A. R., Castera, M. J. T., . . . Delgado, J. (2017). Phase ii trial to evaluate the efficacy and safety of the combination of obinutuzumab and bendamustine treatment in patients (PTS) with relapsed/refractory chronic lymphocytic leukemia (CLL). *Blood, 130*. Retrieved from http://www.embase.com/search/results?subaction=viewrecord&from=export&id=L620334246

Ghia, P., Scarfò, L., Perez, S., Pathiraja, K., Derosier, M., Small, K., . . . Patton, N. (2017). Efficacy and safety of dinaciclib vs ofatumumab in patients with relapsed/refractory chronic lymphocytic leukemia. *Blood, 129*(13), 1876-1878. doi:10.1182/blood-2016-10-748210

Hatake, K., Yamamoto, K., Fukuhara, N., Kusumoto, S., Izutsu, K., Choi, I., . . . Tobinai, K. (2017). Phase 1/2 study evaluating the safety, pharmacokinetics, and preliminary efficacy of venetoclax in Japanese

subjects with chronic lymphocytic leukemia. *Blood, 130*. Retrieved from http://www.embase.com/search/results?subaction=viewrecord&from=export&id=L620333528

Jones, J. A., Robak, T., Brown, J. R., Awan, F. T., Badoux, X., Coutre, S., . . . Owen, C. (2017). Efficacy and safety of idelalisib in combination with ofatumumab for previously treated chronic lymphocytic leukaemia: an open-label, randomised phase 3 trial. *Lancet Haematol, 4*(3), e114-e126. doi:10.1016/s2352-3026(17)30019-4

Lampson, B. L., Kim, H. T., Davids, M. S., Abramson, J. S., Freedman, A. S., Jacobson, C. A., . . . Brown, J. R. (2017). Efficacy results of a phase 2 trial evaluating idelalisib plus ofatumumab in patients with previously untreated chronic lymphocytic leukemia. *Blood, 130*. Retrieved from http://www.embase.com/search/results?subaction=viewrecord&from=export&id=L620334269

Larsen, J. T., Shanafelt, T. D., Leis, J. F., LaPlant, B., Call, T., Pettinger, A., . . . Ding, W. (2017). Akt inhibitor MK-2206 in combination with bendamustine and rituximab in relapsed or refractory chronic lymphocytic leukemia: Results from the N1087 alliance study. *Am J Hematol, 92*(8), 759-763. doi:10.1002/ajh.24762

Mauro, F. R., Carella, A. M., Molica, S., Paoloni, F., Liberati, A. M., Zaja, F., . . . Foa, R. (2017). Fludarabine, cyclophosphamide and lenalidomide in patients with relapsed/refractory chronic lymphocytic leukemia. A multicenter phase I-II GIMEMA trial. *Leuk Lymphoma, 58*(7), 1640-1647. doi:10.1080/10428194.2016.1258698

Ogawa, Y., Izutsu, K., Kiguchi, T., Choi, I., Takatsuka, Y., Ando, K., & Suzumiya, J. (2017). A multicenter, single-arm, Phase II clinical trial of bendamustine monotherapy in patients with chronic lymphocytic leukemia in Japan. *Int J Hematol, 105*(5), 631-637. doi:10.1007/s12185-016-2178-9

Robak, T., Hellmann, A., Kloczko, J., Loscertales, J., Lech-Maranda, E., Pagel, J. M., . . . Jaeger, U. (2017). Randomized phase 2 study of otlertuzumab and bendamustine versus bendamustine in patients with relapsed chronic lymphocytic leukaemia. *British Journal of Haematology, 176*(4), 618-628. doi:10.1111/bjh.14464

Seymour, J. F., Kipps, T. J., Eichhorst, B. F., Hillmen, P., D'Rozario, J. M., Assouline, S., . . . Kater, A. P. (2017). Venetoclax plus rituximab is superior to bendamustine plus rituximab in patients with relapsed/ refractory chronic lymphocytic leukemia-Results from pre-planned interim analysis of the randomized phase 3 murano study. *Blood, 130*. Retrieved from http://www.embase.com/search/results?subaction=viewrecord&from=export&id=L620334274

Stilgenbauer, S., Aktan, M., Coll, C. M. F., Dartigeas, C., Kisro, J., Montillo, M., . . . Leblond, V. (2017). Safety of obinutuzumab alone or combined with chemotherapy in previously untreated (Fit or Unfit) or relapsed/refractory chronic lymphocytic leukemia (CLL) patients: Results from the primary analysis of the phase 3b green study. *Blood, 130*. Retrieved from http://www.embase.com/search/results?subaction=viewrecord&from=export&id=L620334939

Stilgenbauer, S., Chyla, B., Eichhorst, B., Schetelig, J., Munir, T., Hillmen, P., . . . Hallek, M. (2017). Venetoclax in relapsed/refractory chronic lymphocytic leukemia with 17P deletion: Outcome and minimal residual disease from the full population of the pivotal M13-982 trial. *Haematologica, 102*,

311-312. Retrieved from http://www.embase.com/search/results?subaction=viewrecord&from=export&id=L617380020

Thomas, C., Gaballa, S., DiMeglio, M., Alpdogan, S. O., Carabasi, M. H., Chapman, A. E., . . . Weiss, M. A. (2017). Phase I trial study of bendamustine, ofatumumab, and pentostatin (BOP) as salvage regimen for patients with relapsed or refractory non-Hodgkin's lymphoma or chronic lymphocytic leukemia. *Journal of Clinical Oncology, 35*(15). Retrieved from http://www.embase.com/search/results?subaction=viewrecord&from=export&id=L617538743

Wendtner, C. M., Byrd, J. C., Foà, R., Greil, R., Hillmen, P., Jäger, U., . . . Woyach, J. A. (2017). COSMOS: MOR208 plus idelalisib or venetoclax in patients with relapsed or refractory (R/R) chronic lymphocytic leukemia (CLL) or small lymphocytic lymphoma (SLL) previously treated with a Bruton's tyrosine kinase inhibitor (BTKi)-A two-cohort phase II study. *Journal of Clinical Oncology, 35*(15). Retrieved from http://www.embase.com/search/results?subaction=viewrecord&from=export&id=L617434767

Woyach, J. A., Awan, F. T., Jianfar, M., Rogers, K. A., Jones, J., Covey, T., . . . Byrd, J. C. (2017). Acalabrutinib with obinutuzumab in relapsed/refractory and treatment-naive patients with chronic lymphocytic leukemia: The phase 1B/2 ace-cl-003 study. *Blood, 130*. Retrieved from http://www.embase.com/search/results?subaction=viewrecord&from=export&id=L620310965

Zelenetz, A. D., Barrientos, J. C., Brown, J. R., Coiffier, B., Delgado, J., Egyed, M., . . . Hillmen, P. (2017). Idelalisib or placebo in combination with bendamustine and rituximab in patients with relapsed or refractory chronic lymphocytic leukaemia: interim results from a phase 3, randomised, double-blind, placebo-controlled trial. *Lancet Oncol, 18*(3), 297-311. doi:10.1016/s1470-2045(16)30671-4

Zhu, J., Li, J., Zhou, J., Song, Y., Qi, J., Xu, W., . . . Qiu, L. (2017). BGB-3111, a highly specific BTK inhibitor, is well tolerated and highly active in chinese patients with relapsed/refractory B-cell malignancies: Initial report of a phase 1 trial in China. *Blood, 130*. Retrieved from http://www.embase.com/search/results?subaction=viewrecord&from=export&id=L620333446

Burger, J. A., Sivina, M., Jain, N., Kim, E., Kadia, T., Estrov, Z., . . . Keating, M. J. (2018). Randomized trial of ibrutinib versus ibrutinib plus rituximab in patients with chronic lymphocytic leukemia. *Blood*, blood-2018-2010-879429. doi:10.1182/blood-2018-10-879429

Chen, C. I., Paul, H., Le, L. W., Wei, E. N., Snitzler, S., Wang, T., . . . Trudel, S. (2018). A phase 2 study of ofatumumab (Arzerra®) in combination with a pan-AKT inhibitor (afuresertib) in previously treated patients with chronic lymphocytic leukemia (CLL)*. *Leukemia and Lymphoma*, 1-9. doi:10.1080/10428194.2018.1468892

Coutre, S., Choi, M., Furman, R. R., Eradat, H., Heffner, L., Jones, J. A., . . . Davids, M. S. (2018). Venetoclax for patients with chronic lymphocytic leukemia who progressed during or after idelalisib therapy. *Blood, 131*(15), 1704-1711. doi:10.1182/blood-2017-06-788133

Davids, M. S., Hallek, M., Wierda, W., Roberts, A. W., Stilgenbauer, S., Jones, J. A., . . . Seymour, J. F. (2018). Comprehensive safety analysis of Venetoclax monotherapy for patients with relapsed/refractory chronic lymphocytic leukemia. *Clinical Cancer Research, 24*(18), 4371-4379. doi:10.1158/1078-0432.CCR-17-3761

Flinn, I. W., Hillmen, P., Montillo, M., Nagy, Z., Illés, Á., Etienne, G., . . . Stilgenbauer, S. (2018). The phase 3 DUO trial: Duvelisib vs ofatumumab in relapsed and refractory CLL/SLL. *Blood, 132*(23), 2446-2455. doi:10.1182/blood-2018-05-850461

Huang, X., Qiu, L., Jin, J., Zhou, D., Chen, X., Hou, M., . . . Li, J. (2018). Ibrutinib versus rituximab in relapsed or refractory chronic lymphocytic leukemia or small lymphocytic lymphoma: a randomized, open-label phase 3 study. *Cancer Med, 7*(4), 1043-1055. doi:10.1002/cam4.1337

Jones, J. A., Mato, A. R., Wierda, W. G., Davids, M. S., Choi, M., Cheson, B. D., . . . Byrd, J. C. (2018). Venetoclax for chronic lymphocytic leukaemia progressing after ibrutinib: an interim analysis of a multicentre, open-label, phase 2 trial. *Lancet Oncol, 19*(1), 65-75. doi:10.1016/s1470-2045(17)30909-9

Leblond, V., Aktan, M., Ferra Coll, C. M., Dartigeas, C., Kisro, J., Montillo, M., . . . Foa, R. (2018). Safety of obinutuzumab alone or combined with chemotherapy for previously untreated or relapsed/refractory chronic lymphocytic leukemia in the phase IIIb GREEN study. *Haematologica, 103*(11), 1889-1898. doi:10.3324/haematol.2017.186387

Rogers, K. A., Huang, Y., Ruppert, A. S., Awan, F. T., Heerema, N. A., Hoffman, C., . . . Byrd, J. C. (2018). Phase 1b study of obinutuzumab, ibrutinib, and venetoclax in relapsed and refractory chronic lymphocytic leukemia. *Blood, 132*(15), 1568-1572. doi:10.1182/blood-2018-05-853564

Seymour, J. F., Kipps, T. J., Eichhorst, B., Hillmen, P., D'Rozario, J., Assouline, S., . . . Kater, A. P. (2018). Venetoclax-Rituximab in Relapsed or Refractory Chronic Lymphocytic Leukemia. *N Engl J Med, 378*(12), 1107-1120. doi:10.1056/NEJMoa1713976

Chen, C. I., Paul, H., Le, L. W., Wei, E. N., Snitzler, S., Wang, T., . . . Trudel, S. (2019). A phase 2 study of ofatumumab (Arzerra((R))) in combination with a pan-AKT inhibitor (afuresertib) in previously treated patients with chronic lymphocytic leukemia (CLL). *Leuk Lymphoma, 60*(1), 92-100. doi:10.1080/10428194.2018.1468892

1.

Appendix 5 Assessment of risk of bias

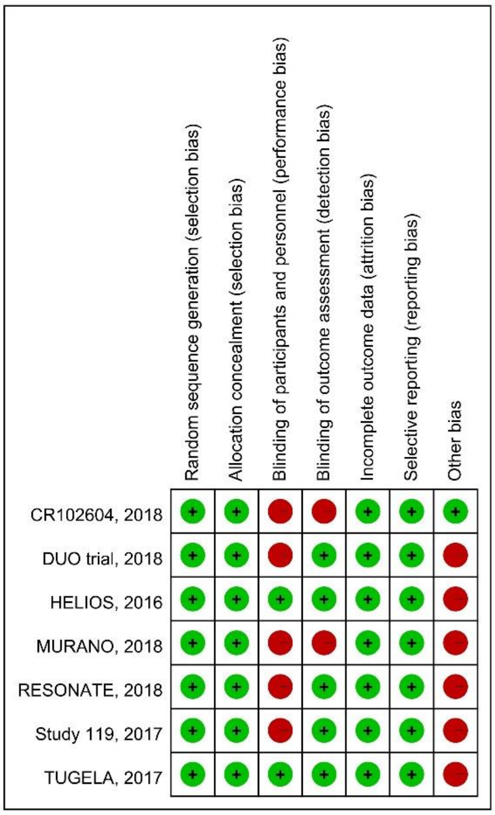

CR102604, 2018[1]

DUO trial, 2018 [2]

HELIOS, 2016 [3]

MURANO, 2018[4]

RESONATE, 2018[5]

Study 119, 2017[6]

TUGELA, 2017[7]

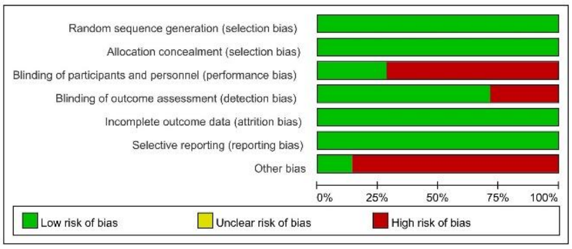

1. Huang, X., et al., *Ibrutinib versus rituximab in relapsed or refractory chronic lymphocytic leukemia or small lymphocytic lymphoma: a randomized, open-label phase 3 study.* Cancer Med, 2018. **7**(4): p. 1043-1055.
2. Flinn, I.W., et al., *The phase 3 DUO trial: duvelisib vs ofatumumab in relapsed and refractory CLL/SLL.* Blood, 2018. **132**(23): p. 2446-2455.
3. Chanan-Khan, A., et al., *Ibrutinib combined with bendamustine and rituximab compared with placebo, bendamustine, and rituximab for previously treated chronic lymphocytic leukaemia or small lymphocytic lymphoma (HELIOS): a randomised, double-blind, phase 3 study.* Lancet Oncol, 2016. **17**(2): p. 200-211.
4. Seymour, J.F., et al., *Venetoclax–Rituximab in Relapsed or Refractory Chronic Lymphocytic Leukemia.* New England Journal of Medicine, 2018. **378**(12): p. 1107-1120.
5. Brown, J.R., et al., *Extended follow-up and impact of high-risk prognostic factors from the phase 3 RESONATE study in patients with previously treated CLL/SLL.* Leukemia, 2018. **32**(1): p. 83-91.
6. Jones, J.A., et al., *Efficacy and safety of idelalisib in combination with ofatumumab for previously treated chronic lymphocytic leukaemia: an open-label, randomised phase 3 trial.* Lancet Haematol, 2017. **4**(3): p. e114-e126.
7. Zelenetz, A.D., et al., *Idelalisib or placebo in combination with bendamustine and rituximab in patients with relapsed or refractory chronic lymphocytic leukaemia: interim results from a phase 3, randomised, double-blind, placebo-controlled trial.* Lancet Oncol, 2017. **18**(3): p. 297-311.

Appendix 6. Efficacy Outcomes for Progression Free Survival (PFS) and Overall Survival (OS) in Network Meta-analysis: Head-to-head comparisons of outcomes

**BR**	0.704 (0.267 to 1.859)	1.931 (0.979 to 3.810)	**1.602 (1.014 to 2.531)**	**1.613 (1.093 to 2.381)**	0.929 (0.348 to 2.482)	0.697 (0.291 to 1.672)	0.861 (0.324 to 2.288)	**2.083 (1.085 to 4.000)**
1.184 (0.608 to 2.305)	**Duv**	**2.742 (1.371 to 5.487)**	2.275 (0.859 to 6.028)	2.291 (0.805 to 6.521)	1.320 (0.715 to 2.437)	0.990 (0.650 to 1.508)	1.223 (0.456 to 3.281)	2.959 (0.919 to 9.530)
**6.155 (3.794 to 9.986)**	**5.200 (3.289 to 8.223)**	**Ibr**	0.830 (0.418 to 1.645)	0.835 (0.382 to 1.828)	**0.481 (0.237 to 0.978)**	**0.361 (0.208 to 0.627)**	**0.446 (0.221 to 0.900)**	1.079 (0.421 to 2.767)
**5.049 (3.785 to 6.733)**	**4.265 (2.176 to 8.360)**	0.820 (0.501 to 1.343)	**IbrBR**	1.007 (0.552 to 1.836)	0.580 (0.217 to 1.555)	0.435 (0.181 to 1.048)	0.538 (0.202 to 1.433)	1.301 (0.586 to 2.885)
**3.030 (2.296 to 4.000)**	**2.560 (1.244 to 5.270)**	**0.492 (0.282 to 0.860)**	**0.600 (0.402 to 0.895)**	**IdeBR**	0.576 (0.200 to 1.658)	0.432 (0.166 to 1.126)	0.534 (0.187 to 1.529)	1.292 (0.604 to 2.761)
**2.367 (1.170 to 4.790)**	**2.000 (1.254 to 3.190)**	**0.385 (0.230 to 0.642)**	**0.469 (0.230 to 0.955)**	0.781 (0.366 to 1.666)	**IdeOfa**	0.750 (0.480 to 1.172)	0.927 (0.342 to 2.514)	2.241 (0.689 to 7.288)
0.616 (0.337 to 1.123)	**0.520 (0.390 to 0.693)**	**0.100 (0.070 to 0.143)**	**0.122 (0.066 to 0.224)**	**0.203 (0.105 to 0.394)**	**0.260 (0.180 to 0.376)**	**Ofa**	1.235 (0.506 to 3.017)	**2.989 (1.003 to 8.902)**
1.108 (0.537 to 2.286)	0.936 (0.461 to 1.899)	**0.180 (0.105 to 0.309)**	**0.219 (0.106 to 0.456)**	**0.366 (0.168 to 0.794)**	**0.468 (0.222 to 0.984)**	1.800 (0.943 to 3.435)	**R**	2.419 (0.747 to 7.832)
**5.882 (4.152 to 8.333)**	**4.969 (2.343 to 10.541)**	0.956 (0.526 to 1.735)	1.165 (0.742 to 1.831)	**1.941 (1.243 to 3.030)**	**2.485 (1.132 to 5.453)**	**9.557 (4.771 to 19.144)**	**5.309 (2.377 to 11.860)**	**VR**

Progression Free Survival | Comparator | Overall Survival

Head-to-head comparisons.

Data are presented as the hazard ratio (95% CI) in the column-defining treatment compared with the row-defining treatment. Comparisons should be read from left to right. Numbers in bold represent statistically significant results.

Ibr, Ibrutinib; IbrBR, Ibrutinib plus Bendamustine Rituximab; VR, Venetoclax Rituximab; IdeOfa, Idelalisib plus Ofatumumab; IdeBR, Idelalisib plus Bendamustine Rituximab; Duv, Duvelisib; B, Bendamustine; R, Rituximab; Ofa, Ofatumumab
